# Influence of magma intrusion on coal geochemical characteristics: a case study of Tiefa Daxing coal mine

**DOI:** 10.1038/s41598-024-58186-5

**Published:** 2024-03-28

**Authors:** Xiang Fu, Xuan Liu, Qixuan Wu, Bin Xiao, Chaojun Fan

**Affiliations:** College of Mining, Liaoning Technical University, Fuxin, 123000 China

**Keywords:** Biogeochemistry, Environmental sciences, Natural hazards

## Abstract

Magma intrusion has an important influence on the physical and mechanical properties of coal and rock. In the area of magma intrusion, disasters such as gas outburst are prone to occur. Revealing its invasion law will be conducive to disaster management and energy development. For this purpose, changes in industrial analysis components of coal, mineral composition, major oxides, trace elements, and rare earth elements of coal under the thermal metamorphism of magma intrusion were analyzed. It is found that the moisture and volatile matter contents of the thermally affected coals in the mining face are generally lower than that of normal coals, while moisture and volatile matter contents are reduced towards to the magma intrusion contact. For example, the moisture and volatile matter of coal sample M01 decreased by 64.6% and 38.6% respectively compared with coal sample M05. During magma intrusion, some minerals remain on the surface of the coal body, resulting in changes in the mineral composition of the coal body. The decrease in carbon atom net spacing, the increase in crystallite aggregation and ductility, and aromaticity in thermally affected coals have a positive impact on the improvement of coal metamorphism. Due to the influences of magmatic intrusion, the variation rules of major oxides in coal are different, and the closer to the magmatic intrusion zone, the easier the major oxides are to be depleted. However, magma intrusion will not lead to the loss of all major oxides in thermally affected coals, such as content of CaO is 54.8%, which is higher than that of coal not affected by magmatic hydrothermal fluid. Most of the trace elements in the thermally affected coals of the No. 9 coal seam are depleted. The contents of rare earth elements are low on the whole coalbasis, with an average of 29.48 μg/g, and the distribution pattern towards to magmatic intrusion shows a wide and gentle “V” curve with left high and right low, showing the characteristics of enrichment of light rare earth elements.

## Introduction

In 2023, Chinese raw coal production and coking raw coal production will be 4.66 billion tons and 1.319 billion tons, respectively, an increase of 2.9% and 5.2% compared with the same period in 2022. China’s metallurgical coal production is relatively stable in each year. From 2011 to 2023, the average metallurgical coal consumption is 275 million tons, and the metallurgical coal production in 2023 is 298 million tons, an increase of 2.82% over the same period in 2022^1–3^. Coal, as the leading energy in China, accounts for more than 60% of the primary energy consumption and will still be the Chinese dominant energy for a long time in the future^4–6^. Chinese coals have the characteristics of wide distribution and large resources, and magma intrusion into the coal seam is also commonly reported from Chinese coalfields^7^. The coal seam invaded by magma has its continuity damaged to a certain extent and its complexity increased, which leads to greater difficulty in mining. In turn, high ash content and poor coal quality greatly reduce its industrial value. The development of microstructure such as pores in coal seams is not conducive to gas closure and increases the risk of coal and gas outbursts^8–10^. The intrusion of magma can not only change the coal grade, adsorption capacity, and pore structure of coal seams but also affect the elemental compositions of coals. Therefore, it is of great practical significance to study the influence of basic magma intrusion on the elements and mineral composition of coals.

Zhang et al.^11^ indicated that contact thermal metamorphism leads to the devolatilization of organic matter in coal and the deposition of pyrolytic carbon, resulting in the increase of Be and Ge content in thermally affected coals near the intrusion. Chen^12^ investigated the magma intruded coal seam and discovered that the SiO_2_ content of the intrusion gradually increased from the bottom to the top. Tang^13^ found that in coal bodies invaded by magma, their porosity increased, their ability to absorb oxygen increased, and they were more susceptible to oxidation. Xu et al.^14^ tested the geochemical composition of coals intruded by magma, and due to the mixed alteration of the magma and coal body, there was little difference in trace elements of coals near the magma intrusion range. Bi^15^ found that the contact metamorphism and thermal evolution of igneous rock on the coal body reduced the volatile matter and moisture contents of the coal body but increased the content of fixed carbon and ash by comparing the industrial analysis and element analysis of normal coals and thermally affected coals. In this paper, we use elemental geochemistry analysis to investigate the impact of magmatic intrusion on the geochemical features of the No. 9 coal seam in the Daxing coal mine.

## Geological setting

**Figure 1:**
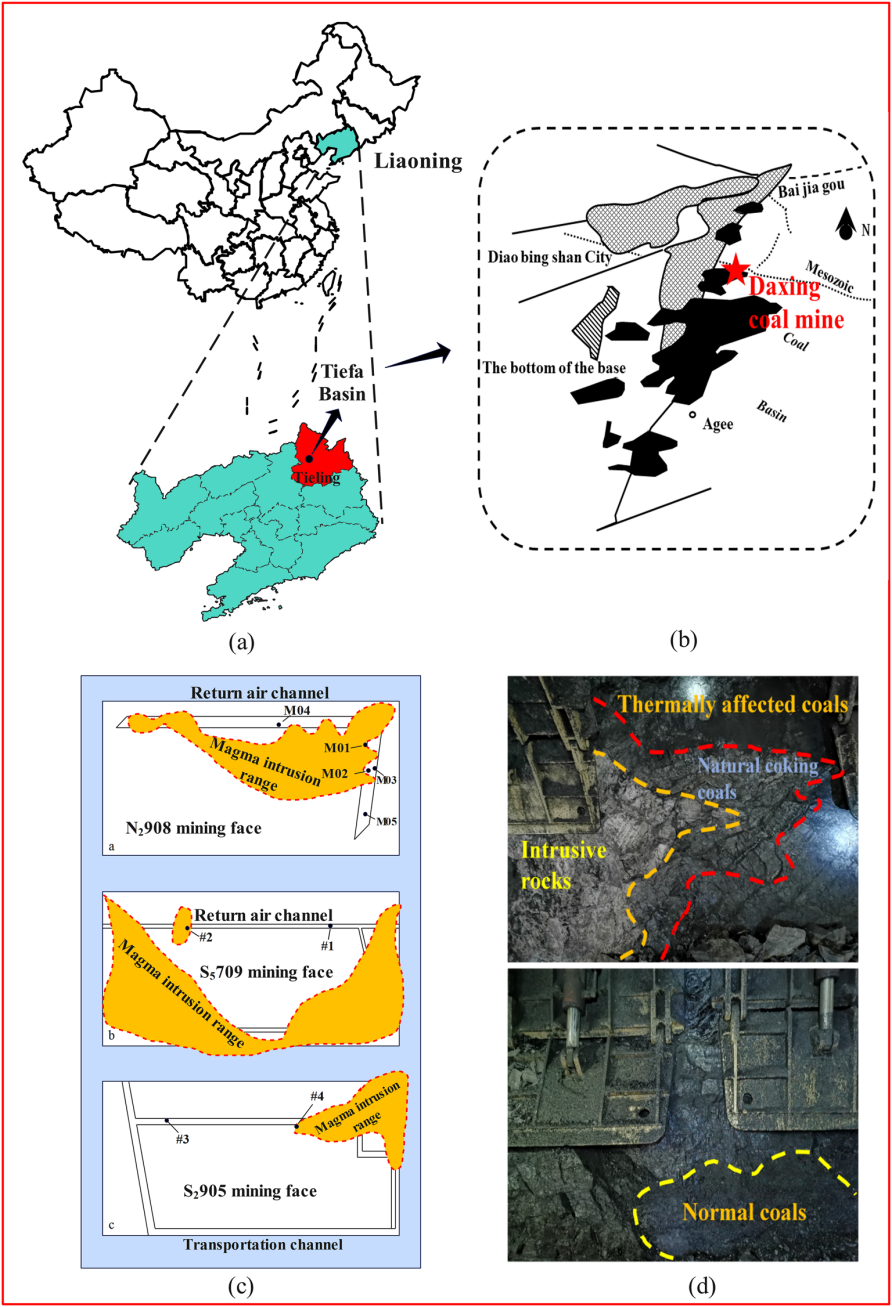
Study area and sampling locations for coal samples. (**a**) Tieling map of Liaoning Province, China; (**b**) Sketch geological map of the Tiefa Basin; (**c**) Magma intrusion zone and sampling point location; (**d**) Site photos of magma intrusion and coal contact.

**Figure 2:**
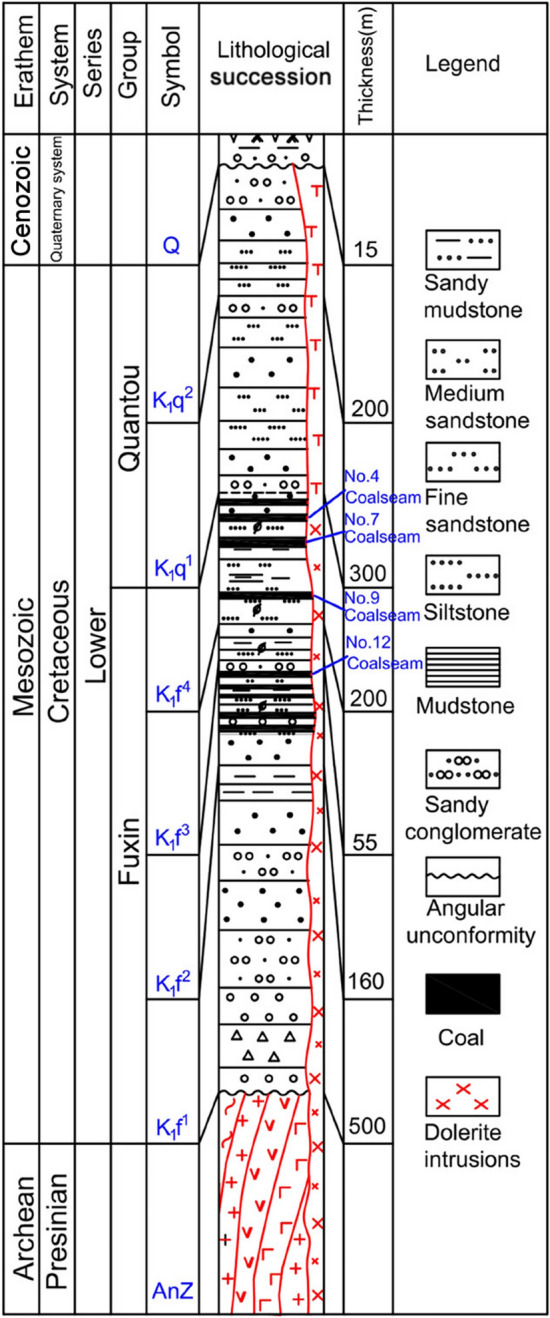
Stratigraphic column of Daxing coal mine.

The Tiefa Basin is located in Diaobingshan City in the north of Liaoning Province (Fig. 1). It is 29.5 km long from north to south, 17.4 km wide from east to west, and covers an area of 513.5 km^2^. Daxing coal mine is located in the southwest of Tiefa Basin, which is controlled by a unilateral oblique fault^16^. The north–south strike of this oblique fault is 6.4 km long, the east–west width is 3.2 km, and the area is 20.48 km^2^. There are many internal faults and some undulating shortaxis anticlines with wavy distribution. The internal stratum of the basin is incomplete, and there is sedimentary discontinuity. The main stratum is the lower Cretaceous Fuxin Formation. This formation is divided into four sections: 1. The bottom sandy gravel section, which is deeply stored, and the lower part are mainly composed of gray, green, and dark brown sandy gravel. The gravels are mainly granite gneiss and quartzite gravels. The upper part is mainly composed of gray and dark gray sandstone, mixed with conglomerate. 2. The lower coal-bearing section is composed of gray black, gray white, gray sandstone, mudstone, coal seam, and carbon mudstone. 3. The middle sandstone and mudstone section is composed of gray, white, and gray fine sandstone mixed with coarse sandstone and mudstone. 4. The upper coal bearing section, which is composed of gray, gray white, and gray black sandstone, mudstone, pebbly sandstone, conglomerate, and coal seam with siderite. The coal bearing stratum is composed of Fuxin Formation of Early Cretaceous age and the only coal-bearing stratum in the coalfield. There are 12 minable coalbeds: the Nos. 2–1, 2–2, 2–3, 3–3, 4–2, 7–2, 8, 9, 9–2, 9–3, 10–1, and 10–2 coals (Fig. 2)^17,18^. The magmatic activity in Daxing coal mine is relatively intense, and its activity form is consistent with the regional situation, with two types: eruption and invasion. The eruption rock is mainly basalt, which appears in the ochre layer of the Cretaceous Sunjiawan Formation and is interbedded with sedimentary rock. The intrusive rocks are mainly diabase. The above two magmatic rocks are distributed in the coal measures, which have a great influence on the coal measures, especially the main coal seams. In addition, the magmatic activity of the coalfield can be divided into three periods. The first stage is the early Jurassic, dominated by volcanic eruptions; the late Cretaceous is the second stage, which is dominated by post-eruption reperfusion. The third activity period is the tertiary period, and diabase intrusion occupies the dominant position of igneous rock intrusion. There is a fair amount of complexity within the igneous rock composition of the N_2_908 mining face. Most of the intrusion horizon can be seen at the top of coal seams 4 and 7^19^. Igneous rock has a thickness of between 28.12 and 50.78 meters^15^.

## Samples collection and experimental methods

The research samples were taken from the N_2_908 mining face of the Daxing coal mine. The N_2_908 mining face is located in the southwest of the North Second Mining Area, with a mining strike length of 977 m and an inclination width of 106 m and 125 m, respectively. The method of sampling of coal seams is in accordance with the national standard GB/T 482-2008. According to the different distance of magma intrusion into the mining face, five coal samples were selected at different positions of N_2_908 mining face, among which coal samples M01-M04 were heat-affected coal and M05 were normal coal. In addition, the coals of mining face S_5_709 and S_2_905 were also collected. Four coal samples were selected in the position that was completely unaffected by magma and the magma intrusion area, which were recorded as S_5_709 normal coal (# 1), S_5_709 metamorphic coal (# 2), S_2_905 normal coal (# 3), and S_2_905 metamorphic coal (# 4). The weight of each coal sample is 2 kg^15,20^. After the coal sample is mined, it is sealed in a sealed bag and transported to the laboratory. The coal sample is sieved into the particle size required for the experiment by mechanical crushing. After screening, the experimental coal samples with the required particle size are obtained. In order to eliminate the influence of external moisture on the coal spontaneous combustion experiment, the appropriate quality of coal samples were put into the vacuum drying box, the temperature of the drying box was adjusted to 40 °C, and the coal samples were dried for 24 h in the same vacuum environment. After the drying, the coal samples were put into the self-sealing bag to prevent the water vapor and oxygen in the air from contacting the coal samples, thus affecting the experimental results.

The standard proximate analysis (moisture, ash yield, volatile matter content, and fixed carbon) of coal samples was completed at Liaoning Technical University. After the coal samples were mechanized and crushed, the coal samples with a particle size of 80–200 µm were selected as the research object, and the 5E-MAG6600B automatic industrial analyzer was used for testing.

The analysis of major oxides, trace elements, and rare earth elements of coal samples was completed by Wuhan Shangpu Analytical Technology Co., Ltd. in China. The ZSX Primus II wavelength dispersive X-ray fluorescence spectrometer (XRF) produced by Rigaku was used for determining major oxide contents, including SiO_2_, TiO_2_, Al_2_O_3_, Fe_2_O_3_, MnO, MgO, CaO, K_2_O, Na_2_O, P_2_O_5_^21^. The analysis procedure complies with the national standard GB/T14506.28-2010. Trace elements and rare earth elements in raw coals are analyzed by inductively coupled plasma mass spectrometry (ICP-MS). First, 0.500 mL (1 + 1) of HNO_3_ and 1.00 ml of HF were used to digest 25.0 mg of rock powder (200 mesh) in a firmly closed Teflon screw-cap beaker. The dried sample was digested once again using 0.500 mL (1 + 1) of HNO_3_ and 1.50 ml of HF after evaporation, and it was then dried once more (1 + 1) HNO_3_ was then added to the sample at a volume of 2.00 mL. After drying the mixture once again, the process was repeated while adding HNO_3_. For trace element analysis, the solution was finally diluted with 1.00% HNO_3_ to 50.0 mL. The analysis procedure conformed to the Chinese national standard GB/T14506.30-2010. The XRD testing was conducted using a German Brooke D8 ADBANCE X-ray diffractometer for phase analysis. The collected coal samples were mechanically crushed and ground, and the samples to be tested were obtained after 300 mesh sieve. The test conditions are as follows: X-ray tube, Cu target, Ka radiation sampling, test voltage and current are 40 kV and 30 mA respectively; the scanning speed is set to 0.1 s/step, and the sampling interval is 0.019450.

## Results and discussions

### Effect of magma intrusion on standard coal quality features

The moisture (%), ash yield (%), volatile matter (%), and fixed carbon (%) contents of each coal sample are shown in Table 1. By comparing and analyzing five coal samples from the N_2_908 mining face, it was determined that the closer the coal is to the range of igneous intrusion, the lower its moisture content. This could be due to the high temperature that accompanied the igneous intrusion, which caused the gasification of moisture in the coal body. However, the ash content increases with the decrease of the magma intrusion range, and it can be concluded that the decrease of the distance from the magma intrusion body will lead to the enhancement of contact thermal metamorphism. The volatile matter content diminishes with the decrease of the magma intrusion range, which indicates that the metamorphic degree of the coal body invaded by magma increases. Hence, the content of fixed carbon tends to be higher than that of normal coal in thermally affected coal, and the intrusion of magma is also obtained from this perspective, which will promote the metamorphism of coal.

**Table 1:** Results of industrial analysis of various coal samples (ad: air-dry basis).

Mining areas	Coal samples	*M*_ad_/%	*A*_ad_/%	*V*_ad_/%	*F*_Cad_/%	*R*_max_/%
N_2_908	M01	1.58	16.36	27.55	64.73	1.13
N_2_908	M02	1.74	16.29	28.24	60.48	1.05
N_2_908	M03	2.71	10.24	33.28	59.07	0.71
N_2_908	M04	2.78	9.07	34.74	56.95	0.63
N_2_908	M05	4.46	6.82	44.85	44.11	0.54
S_5_709	#1	5.04	6.91	36.75	51.30	0.59
S_5_709	#2	2.76	12.38	24.60	60.26	1.27
S_2_905	#3	3.43	8.44	35.16	52.97	0.55
S_2_905	#4	1.58	13.03	27.77	57.62	1.09

### Effects of magma intrusion on mineralogical composition

**Figure 3:**
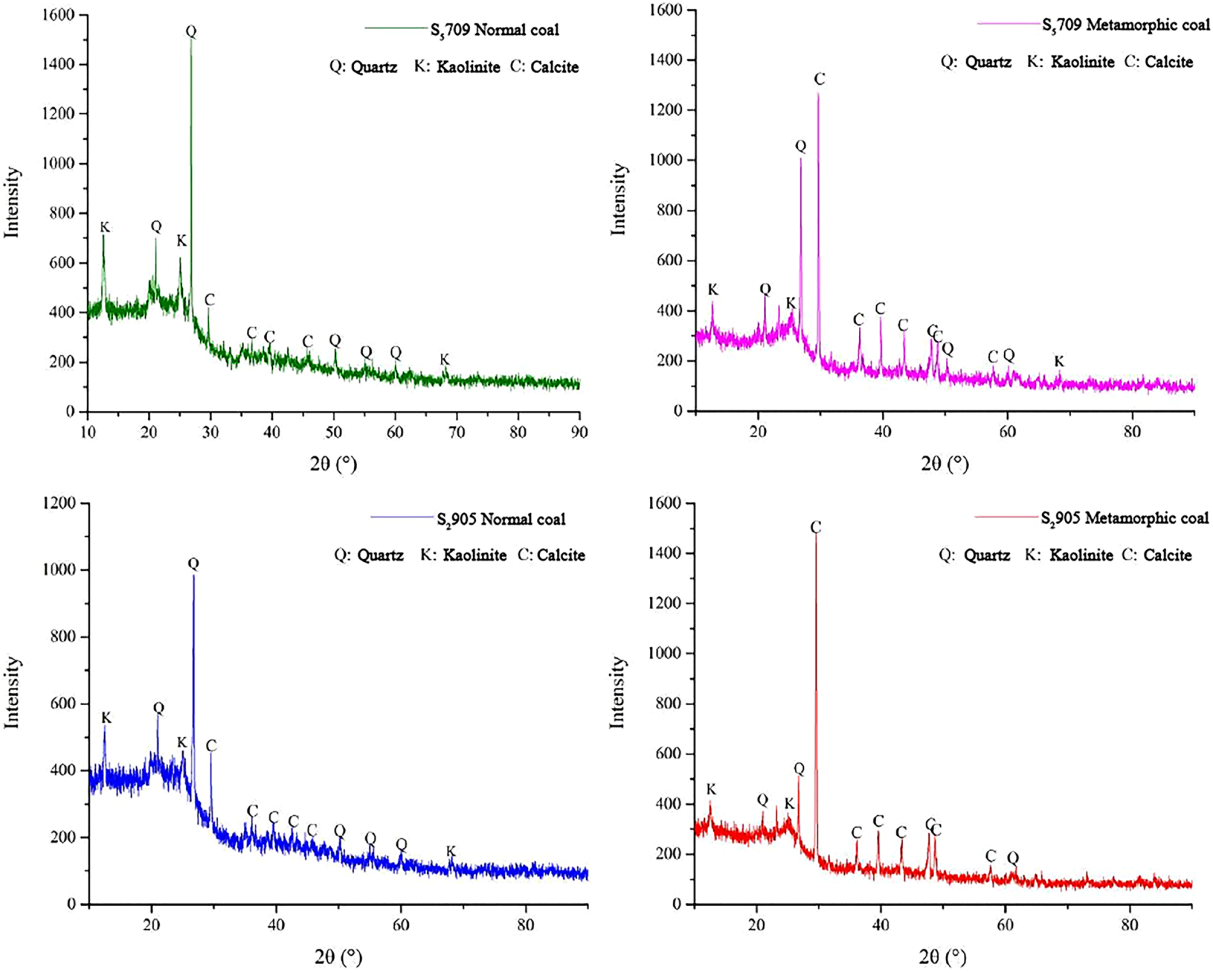
XRD spectra of normal and thermally affected coals.

**Figure 4:**
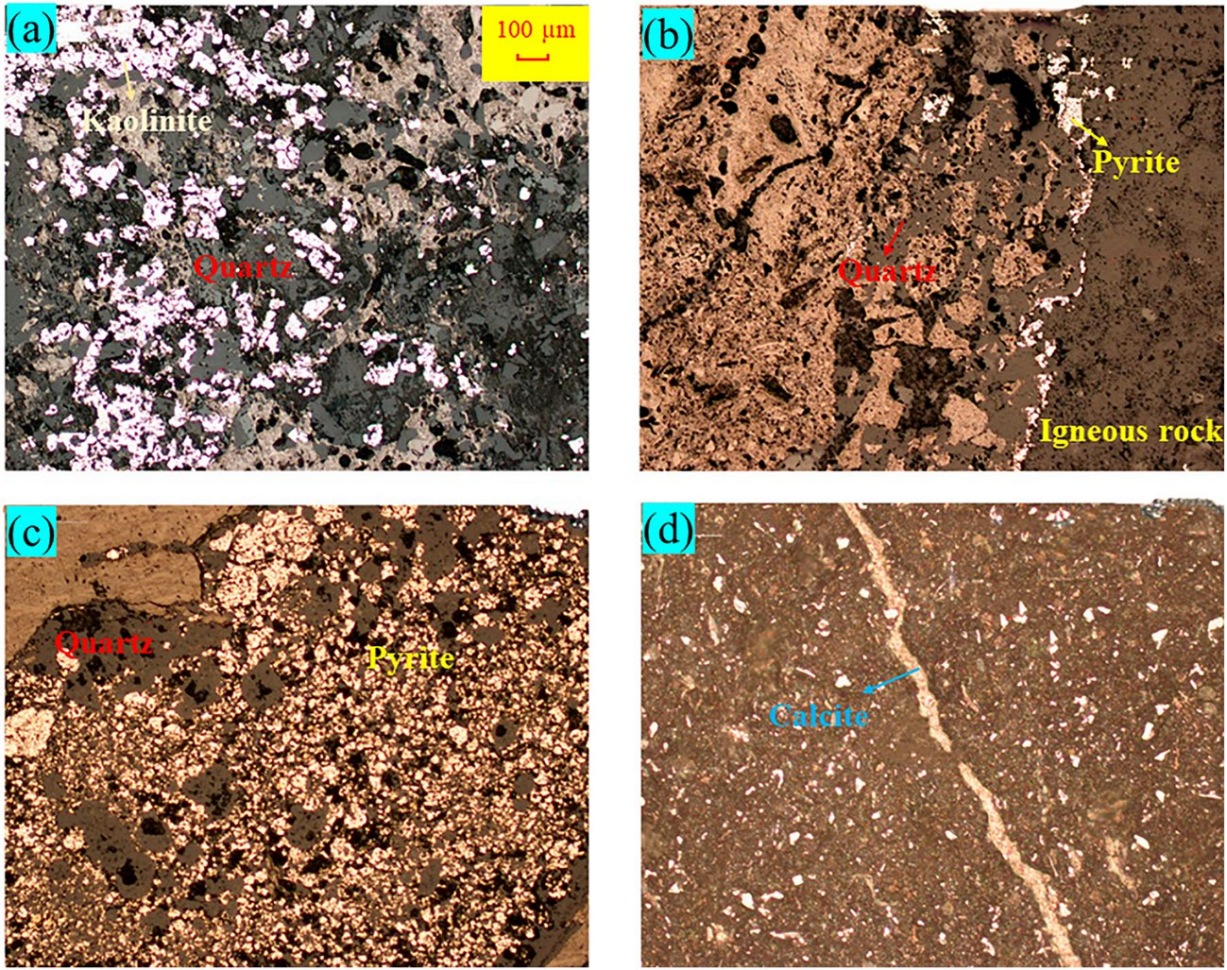
Microscopic images of minerals in normal and thermally affected coals. (**a**) N_2_908 mining face normal coals; (**b**) N_2_908 mining face thermally affected coals; (**c**) S_5_709 mining face thermally affected coals; (**d**) S_2_905 mining face thermally affected coals.

The mineral composition of coal seams intruded by magma depends on the chemical composition and crystallization conditions of magma, which is of great significance for understanding the influence of magma intrusion on coal geochemical characteristics. The minerals in the Daxing coal mine are mainly quartz and clay minerals and contain amounts of other minerals such as calcite and pyrite (Figs. 3 and 4). It also can be seen that the normal coals in S_5_709 and S_2_905 mining faces contains more quartz and only some kaolinite and calcite, while the calcite contents of thermally affected coals in S_5_709 and S_2_905 mining faces affected by magma intrusion significantly increases. Chen et al.^22^ and Dai et al.^23^ found that magmatic hydrothermal solution contains exogenic minerals such as calcite and pyrite, which is basically consistent with the results of this paper. It should be noted that the contents of calcite in thermally affected coals have increased significantly.

As magma intrusion is accompanied by extremely high temperatures, the mineralogical compositions of coal could be changed. There are obvious differences in the diffraction peak spectra of minerals in normal coal and thermally affected coal. When magma infiltrates into the coal seam, some minerals will remain on the surface of the coal body, thus changing the composition of the coal body. The diffraction peak intensities of normal coal, quartz, and kaolinite in S_5_709 and S_2_905 mining faces in the study area are the highest, while other components are relatively low, indicating that these two minerals are the main components of normal coal, with a small amount of calcite. However, in thermally affected coal, the diffraction peak intensity of calcite has been greatly improved, especially in the thermally affected coal S_2_905 mining face (# 4), with a significant increase, while the diffraction peak intensity of quartz and kaolinite has been significantly reduced.

To investigate the influences of magma intrusion on the mineral composition of coals, we analyzed the XRD spectra based on prior researches and presented the relative mineral contents of unaffected and thermally affected coals in Table 2. Table 2 shows that the normal coal sample (# 1) from the S_5_709 mining face contained 41.5% quartz, 55.6% kaolinite, and only 2.9% calcite. The thermally affected coal sample (# 2) from the S_5_709 mining face showed a significant decrease in quartz and kaolinite contents after exposure to magma intrusion, while the calcite contents increased significantly to 52.5%. The normal coal sample (# 3) from the S_2_905 mining face contained similar levels of quartz and kaolinite at 46.8% and 38.1%, respectively, and a calcite contents of 15.1%. Conversely, the thermally affected coal sample (# 4) demonstrated a significant increase in calcite contents to 61.4% and a significant reduction in quartz contents to 10.8%.

**Table 2:** Mineralogical compositions of coal samples from S_5_709 and S_2_905 mining faces.

Coal samples	Relative contents/%		
	Quartz	Kaolinite	Calcite
#1	41.5	55.6	2.9
#2	27.2	20.2	52.5
#3	46.8	38.1	15.1
#4	10.8	27.8	61.4

**Figure 5:**
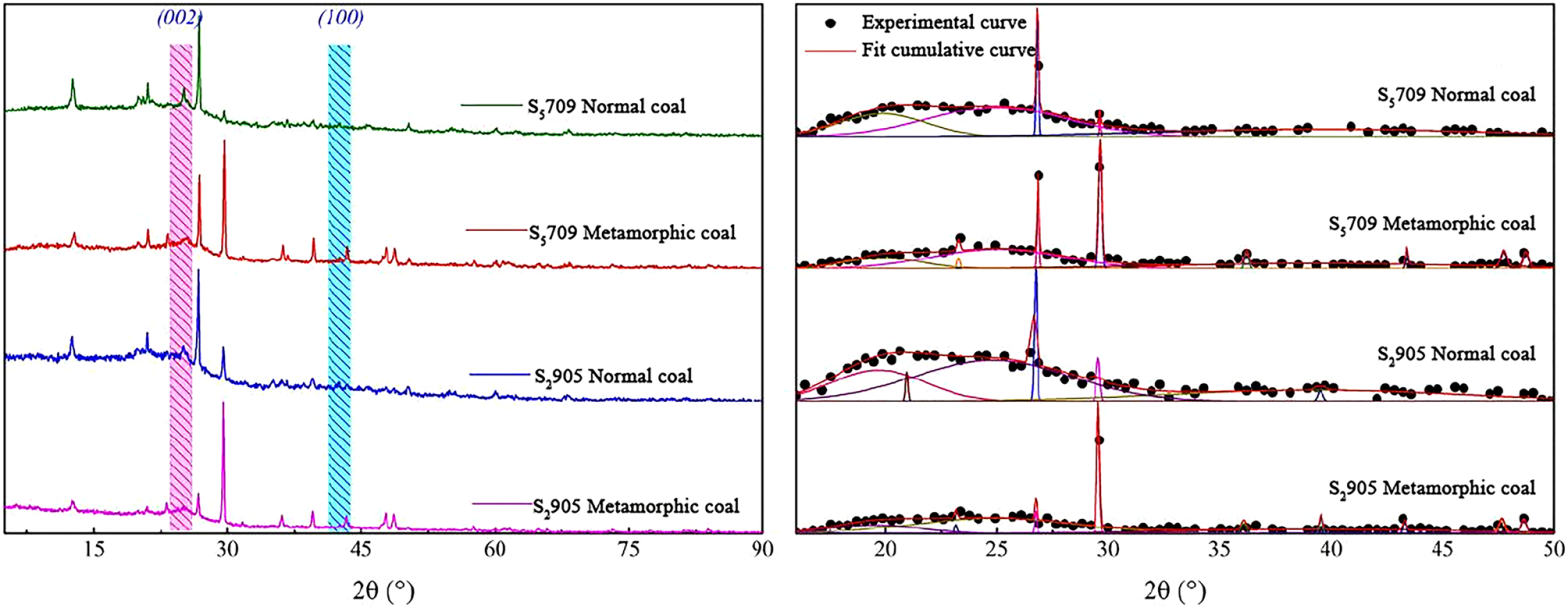
Curve fitting of the peaks of coal samples from 16° to 50°.

In order to further explore the influences of magma intrusion on coal structure, the 16–50° spectral region corresponding to 2θ was fitted, and the results were shown in Fig. 5. It can be seen that the diagrams of the four coal samples correspond to the (002) peak and (100) peak at ~ 25° and ~ 40° respectively, and are much more obvious than the peaks in other positions, indicating they are related with coaly material or organic matter. The peak of (002) is the superposition of (002) band and *γ* band, which is related to the stacking of aromatic ring layers in coal. The (100) peak is attributed to the degree of condensation of the aromatic ring, that is, the size of the aromatic carbon network in the coal.

The structural parameter analysis of XRD for normal coals and thermally affected coals were shown in Table 3. Compared with normal coals and thermally affected coals, it was found that the carbon source net spacing (*d*_002_) of metamorphized coal decreases, while the stacking degree (*L*_*c*_) and ductility (*L*_*a*_) of microcrystalline increases and the aromatic *f*_*a*(*XRD*)_ also increase, indicating that the degree of metamorphism of the coal body increases due to the intrusion of magma.

**Table 3:** Analysis of XRD structural parameters of sample coals.

Coal samples	*d*_*002*_	*L*_*c*_	*L*_*a*_	*f*_*a*(*XRD*)_
#1	3.557	11.87	12.29	0.67
#2	3.553	12.60	12.84	0.75
#3	3.586	10.48	13.67	0.69
#4	3.569	10.89	14.46	0.77

### Geochemical compositions

#### Major oxides

We used the ZSX Primus II wavelength dispersive X-ray fluorescence spectrometer (XRF) produced by Rigaku was used for determining major oxide contents in coal samples. The results are shown in Table 4. Sample M01, which was affected by magma intrusion, exhibited high proportions of CaO, SiO_2_, Fe_2_O_3_, and Al_2_O_3_, at 54.8%, 27.85%, 8.57%, and 5.60%, respectively. Together, these oxides account for 96.82% of the total major oxides contents. SiO_2_ is the dominant major oxide in normal coals, while the content of SiO_2_ in coals affected by heat were different, indicating that the intrusion of magma has little effect on the content of SiO_2_. This is mainly because the SiO_2_ in coals mainly came from the contribution of quartz and kaolinite, which mainly existed in the form of Mosaic in coals, and it was difficult to move in the form of fluid during the later magma intrusion and heating process. The significant increase in CaO contents implies that magma intrusion into the coal seam results in CaO enrichment. Coal sample M02, which was less influenced by magma intrusion than coal sample M01, exhibits the highest contents of SiO_2_ among its major oxides, at 57.35%. Additionally, the contents of Al_2_O_3_, Fe_2_O_3_, CaO, and K_2_O are higher, and the combined contents of these five major oxides account for 96.87% of the total contents. Coal sample M03 has a main element contents of SiO_2_, Al_2_O_3_, and Fe_2_O_3_, which together account for 91.01% of the total. This may be related to migmatic minerals mixed with coaly material. However, compared to other thermally affected coals and normal coals, the Fe_2_O_3_ contents were significantly higher. This implies that magma intrusion was not the direct cause of the increase in Fe_2_O_3_ contents in coal sample M03. Coal sample M04 has major oxides consisting mainly of SiO_2_, Al_2_O_3_, Fe_2_O_3_, and CaO, which together account for 95.36% of the total. Normal coal sample M05 has SiO_2_, Al_2_O_3_, Fe_2_O_3_, MgO, and K_2_O contents of 57.29%, 22.08%, 9.32%, 3.18%, and 3.18%, respectively, which together account for 95.05% of the total. As the contents of SiO_2_ are higher than those of Al_2_O_3_, it implies that quartz provided excess silicon compared to the silicon contents of kaolinite.

**Table 4:** The contents of the major oxides of coals in the Daxing coal mine.

Coal samples	Quality fractions/%									
	SiO_2_	TiO_2_	Al_2_O_3_	Fe_2_O_3_	MnO	MgO	CaO	Na_2_O	K_2_O	P_2_O_5_
M01	27.85	0.27	5.60	8.57	0.47	1.26	54.80	0.27	0.82	0.09
M02	57.35	0.81	15.09	5.74	0.22	1.49	16.33	0.50	2.36	0.10
M03	42.58	0.57	11.74	36.69	0.90	1.68	2.41	0.72	2.01	0.71
M04	31.03	0.53	10.65	13.97	0.33	1.78	39.71	0.54	1.32	0.13
M05	57.29	1.60	22.08	9.32	0.07	3.18	1.46	1.58	3.18	0.24
Average value	43.22	0.76	13.03	14.86	0.40	1.88	22.94	0.72	1.94	0.25

In addition, it can be analyzed in Table 4 that the contents of major oxides in normal coals and thermally affected coals are quite normal. For coal sample M05, the contents of SiO_2_, TiO_2_, Al_2_O_3_, MgO, Na_2_O and K_2_O are higher than in coal samples M01-M04, which indicates that the intrusion of magma may lead to the loss of these major oxides in thermally affected coal samples. The contents of Fe_2_O_3_, MnO, CaO and P_2_O_5_ in thermally affected coals are higher than those in normal coals, which may be the result of thermal contact metamorphism during magmatic intrusion. Fe_2_O_3_, MnO, and P_2_O_5_ show the same change mode, indicating that they have the same source and occurrence state. The contents of SiO_2_, TiO_2_, Al_2_O_3_, MgO, Na_2_O, K_2_O, and P_2_O_5_ in thermally affected coal sample M01, which is the most obviously intruded by magma, are lower than that in other thermally affected coals, indicating that the closer the coal is to the magmatic intrusion zone, the easier it is to lose its major oxides, while the variation pattern of the major oxides contents in coal far from the magma intrusion zone are not obvious.

#### Trace elements

We used inductively coupled plasma mass spectrometry (ICP-MS) to analyze trace elements in coals. Table 5 shows the average contents of trace elements in the coal of the Daxing coal mine and in the crust of Chinese coals and world coals. The enrichment coefficient is usually used to evaluate the enrichment degree of trace elements. Dai et al.^24^ proposed an evaluation index method for the enrichment of trace elements in coal. By calculating the enrichment coefficient of trace elements in coal samples (CC is the ratio of trace elements in the studied samples to the world or Chinese coals average), they can be divided into the following six categories: abnormal enrichment (CC > 100), high enrichment (10 < CC < 100), enrichment (5 < CC < 10), slight enrichment (2 < CC < 5), normal (0.5 < CC < 2), and deficit (CC < 0.5)^25–28^.

**Table 5:** The trace element contents of coal samples from the Daxing coal mine.

Coal samples	Trace elements mass fraction /(µg/g)								
	M01	M02	M03	M04	M05	Average values	Crust	Chinese coals	World coals
Be	0.42	1.02	0.52	0.94	1.38	0.85	2.80	2.11	1.60
Sc	1.48	1.80	0.80	0.92	16.5	4.31	22.00	4.38	3.90
V	11.9	13.4	6.44	7.72	131	34.0	135.0	35.10	25.00
Cr	10.5	10.2	4.52	5.60	98.7	25.9	100.0	15.40	16.00
Co	3.72	4.29	3.53	2.68	13.6	5.56	25.00	7.08	5.10
Ni	6.08	5.97	5.12	6.24	38.9	12.46	75.00	13.70	13.00
Cu	6.96	7.79	3.63	5.05	68.3	18.34	55.00	17.50	16.00
Zn	24.0	6.41	18.7	9.17	122	36.1	70.00	41.40	23.00
Ga	2.45	2.57	1.44	1.27	21.8	5.90	15.00	6.55	5.80
Rb	17.0	18.5	7.28	6.83	123	34.5	90.00	9.25	14.00
Sr	50.7	87.6	179	172	360	169.8	375.0	140.00	110.00
Zr	21.7	23.0	11.0	12.1	73.9	28.3	165.0	89.50	36.00
Nb	1.82	1.92	0.97	1.03	16.0	4.34	20.00	9.44	3.70
Cs	1.30	1.55	0.58	0.62	11.6	3.12	3.00	1.13	1.00
Ba	67.3	71.3	106	147	469	172.3	425.0	159.0	150.0
Hf	0.53	0.67	0.31	0.32	2.07	0.78	3.00	3.71	1.20
Ta	0.12	0.15	0.06	0.063	1.18	0.32	2.00	0.62	0.28
Tl	0.12	0.18	0.26	0.25	0.68	0.30	0.45	0.47	0.63
Pb	2.14	2.50	2.42	4.93	24.8	7.35	12.50	15.10	7.80
Th	1.43	1.88	0.73	0.78	14.0	3.77	9.60	5.84	3.30
U	0.54	0.44	0.21	0.26	5.11	1.31	2.70	2.43	2.40

**Figure 6:**
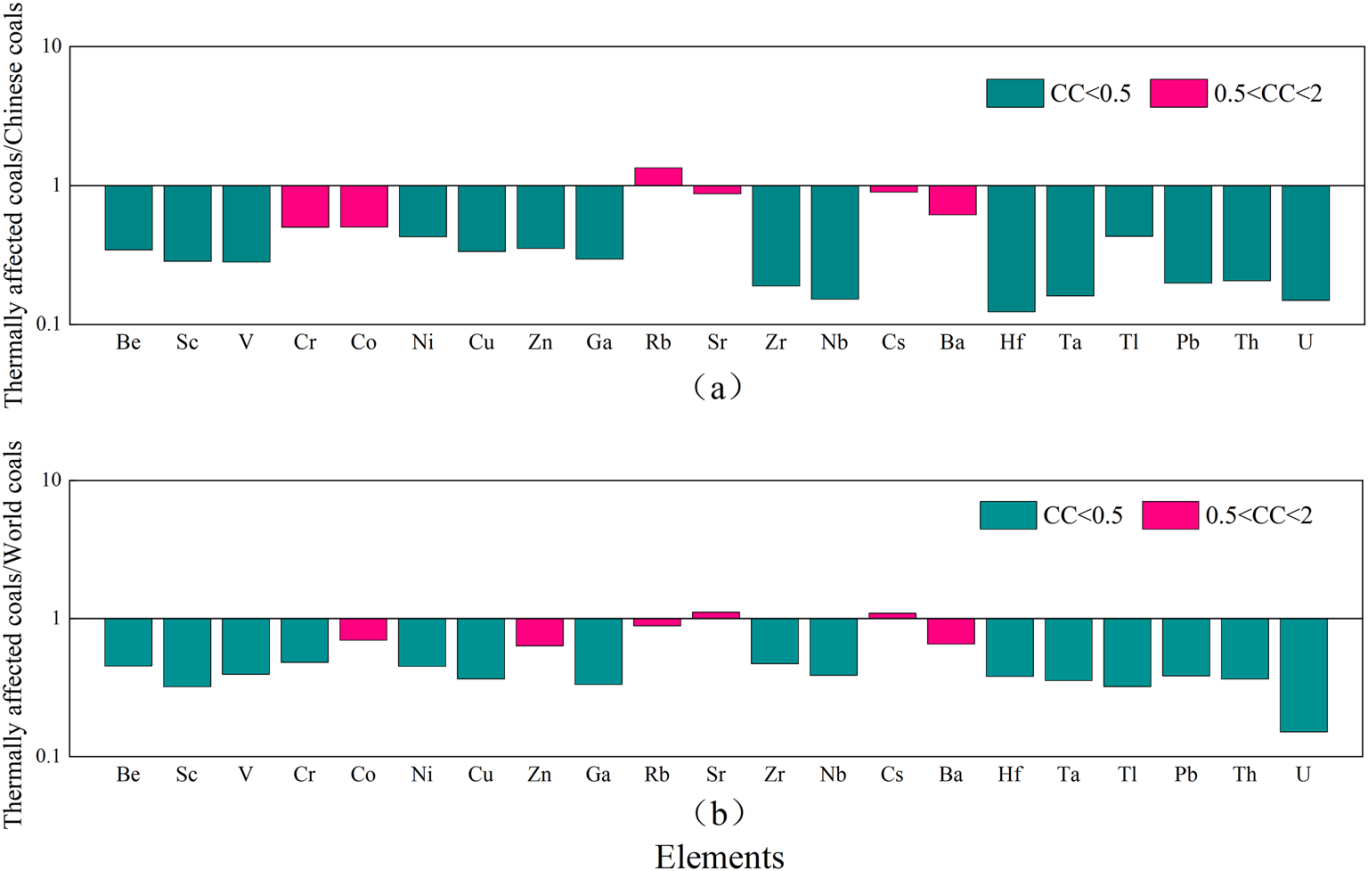
Comparison of trace elements in thermally affected coals from Daxing coal mine and Chinese coals, world coals. (**a**) Thermally affected coals/Chinese coals; (**b**) Thermally affected coals/world coals.

**Figure 7:**
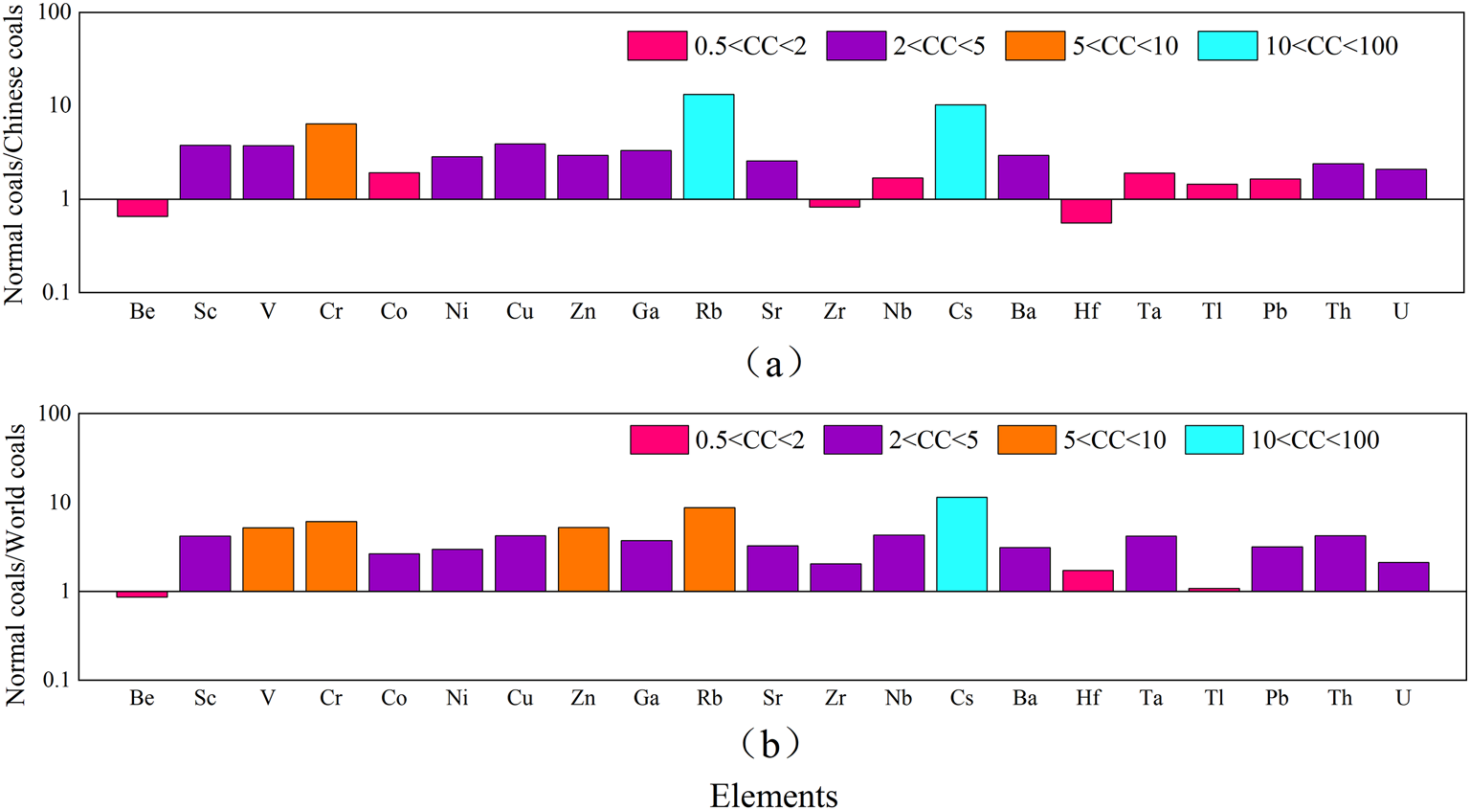
Comparison of trace elements in normal coals from Daxing coal mine and Chinese coals, world coals. (**a**) Normal coals/Chinese coals; (**b**) Normal coals/world coals.

In order to conveniently describe the enrichment degree of trace elements in coals, the contents of trace elements in thermally affected coals and normal coals, Chinese coals, and world coals in the Daxing coal mine coal samples are compared and analyzed based on the enrichment coefficient 1. The results are shown in Figs. 6 and 7. In Fig. 6, the turquoise indicator indicates that the element enrichment coefficient in coal is depleted (CC < 0.5), and the red indicator indicates that it is close to the world and Chinese coals averages (0.5 < CC < 2). In Fig. 6a, by comparing the contents of trace elements in thermally affected coals and Chinese coals, it is found that, except for those elements of Cr, Co, Rb, Sr, Cs, and Ba, which are normal, other elements are depleted. By comparing the contents of trace elements in thermally affected coals and world coals in Fig. 6b, it is determined that, except for Co, Zn, Rb, Sr, Cs, and Ba elements, which are normal, other elements are deficient, which is close to the analysis result in Fig. 6a. Through the above analysis, it is found that magmatic intrusion makes most of the elements in the coal loss, which is mainly because during the coalification process, the contents of trace elements in the coals will be greatly affected by the intrusion of magmatic hydrothermal solution. According to general acceptance, after the intrusion of heavy metal rich hydrothermal solution into the coal seam, it moves and diffuses along the fractures to the surrounding rock, and the carried trace elements are precipitated under appropriate conditions, or absorbed by clay minerals or organic matter in the coals. Therefore, the element loss in coals could develop^29^. In Fig. 7, purple indicates slight enrichment (2 < CC < 5), orange indicates enrichment (5 < CC < 10), and blue indicates high enrichment (10 < CC < 100). In Fig. 7a, by comparing the contents of trace elements in normal coals and Chinese coals, it is found that Be, Co, Zr, Nb, Hf, Ta, Tl, and Pb are normal, while Cr is enriched, Rb is highly enriched, and other elements are slightly enriched. In Fig. 7b, by comparing the contents of trace elements in normal coals and world coals, it is found that Be, Hf, and Tl are close to average values, while the elements of V, Cr, Zn, and Rb are enriched, with Cs being highly enriched, and other elements are slightly enriched. Based on the above analysis, it has been determined that most trace elements in the coals not affected by magmatic intrusion are slightly enriched, and some elements are highly enriched.

In general, magmatic intrusion will have a certain impact on the occurrence of trace elements in coals and make them migrate or enrich. Most of the trace elements in the thermally affected coals intruded by magma in the Daxing coal mine are depleted. Only a few trace elements are normal, while most of the trace elements in the normal coals are slightly enriched.

Different trace elements in coals will have different effects on the environment. Wu et al.^30^ have classified trace elements in coal according to the degree of their potential environmental impact. Although there are some differences in definitions, most of them include ten typical environmentally sensitive trace elements in coals, such as Be, Cr, Mn, Co, Ni, As, Tl, Cd, U, Hg, and Pb^31^.

**Figure 8:**
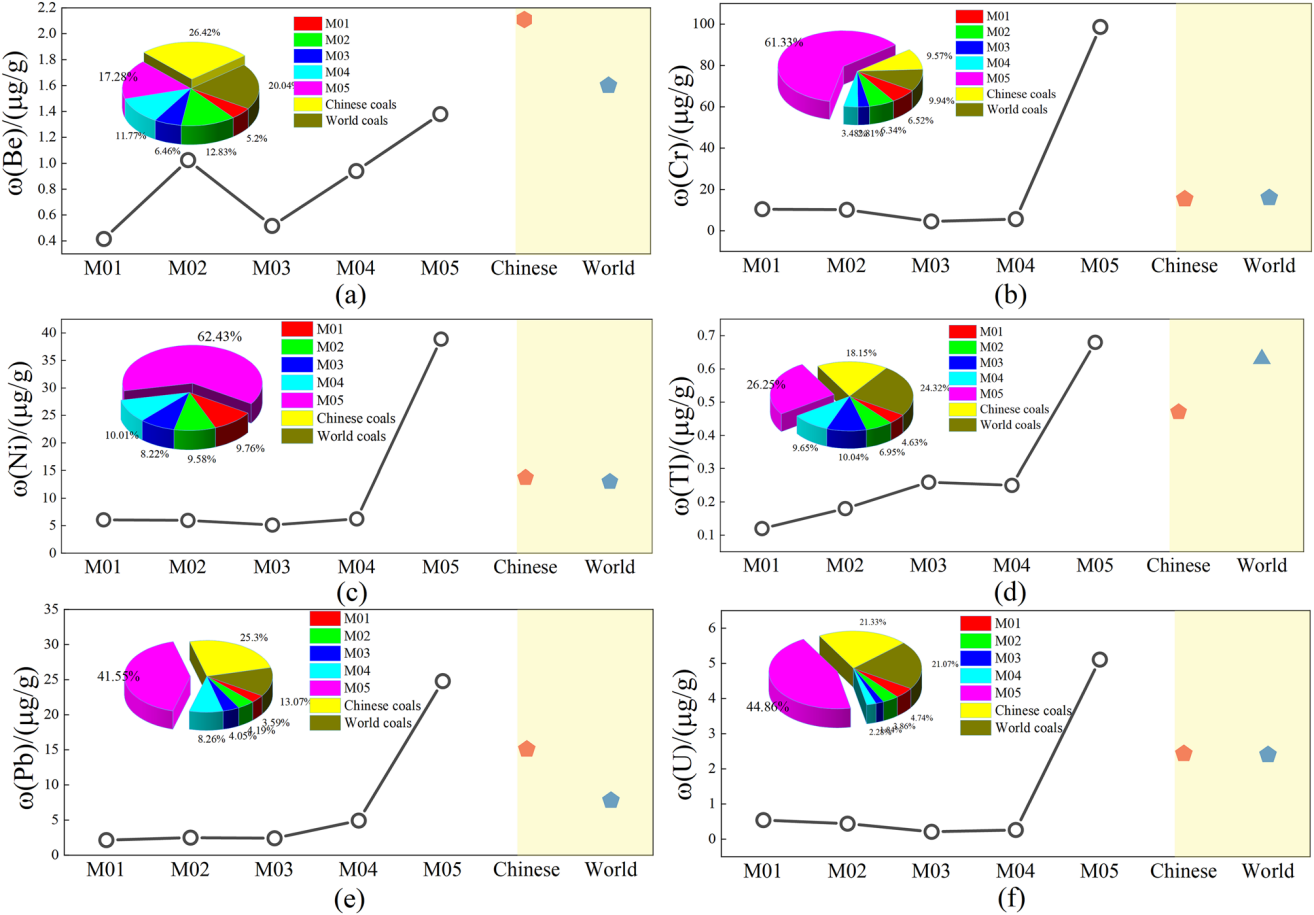
Comparison of potentially hazardous trace elements with Chinese and world coals averages. (**a**) Be; (**b**) Cr; (**c**) Ni; (**d**) Tl; (**e**) Pb; (**f**) U.

In order to study the influences of magmatic intrusion on the contents change of potentially hazardous trace elements in coal, trace elements in six kinds of coal, as shown in Fig. 8 below, were selected for analysis. Among them, the Be element content of thermally affected coals are between 0.42 and 1.02 µg/g, with an average of 0.36 µg/g. The thermally affected coal sample M01, which is greatly affected by mag-matic intrusion, has the lowest Be element content, while the normal coal sample M05, which is farthest from the magmatic intrusion zone, has the highest Be element content. However, it can be seen from Fig. 8a that the Be element content of five coal samples is lower than that of Chinese coals and world coals average. The content of Cr element in thermally affected coals is between 4.52 and 10.5 µg/g, with an average of 7.71 µg/g. In coal samples M01–M04, the content of Cr element is similar. Due to the affected of magmatic intrusion, the content of Cr element is lower than that of Chinese coals and world coals averages. In coal sample M05, the content of Cr element is higher than that of other coal samples and Chinese coals and world coals, indicating that magmatic intrusion leads to the reduction of Cr element content in thermally affected coals. The content of Ni in thermally affected coals ranges from 5.12 to 6.24 µg/g, with an average of 5.85 µg/g, and its distribution characteristics are similar to that of Cr. The content of Tl element in thermally affected coals ranges from 0.12 to 0.26 µg/g, with an average of 0.2 µg/g. It can be seen from Fig. 8d that the content of Tl element in coal sample M05 is obviously higher than that in thermally affected coals and Chinese coals, which is close to that in world coals. Pb in metamorphic coal ranges from 2.14 to 4.93 µg/g, with an average of 2.99 µg/g. The content of U element in thermally affected coals ranges from 0.21 to 0.54 µg/g, with an average of 0.36 µg/g, and the distribution characteristics are similar to those of Cr and Ni elements. Among them, the contents of medium volatile and nonvolatile elements (Cr, Ni, etc.) are relatively high. Due to the affected of magma intrusion, the average contents of medium volatile volatile elements (Pb, Tl, etc.) in unaffected coal are generally less than 10 µg/g, which are generally low. The content of Tl element in coal sample M01 is only 0.12 µg/g.

In general, compared with Chinese coals and world coals affected by magmatic intrusion, the contents of potentially hazardous trace elements in thermally affected coals of the Daxing coal mine are lower than that of Chinese coals and world coals averages, and the contents of Cr, Ni, Tl, Pb, and U in normal coal are much higher than that of thermally affected coals. Among them, the contents of Be, Tl, and Pb all increase with the decrease in distance from the magmatic intrusion zone. Except for coal sample M05, the contents difference in other coal samples are relatively small, indicating that the magmatic intrusion has a tendency to dilute and reduce the contents of potentially hazardous trace elements in unaffected coal samples but the impact on different coal samples are slightly different.

#### Rare earth elements

According to the similarities and differences in the geochemical properties of rare earth elements (REE), they can be divided into the following three categories: light rare earth elements (LREE), including La, Ce, Pr, and Nd; medium rare earth elements (MREE), including Sm, Eu, Gd, Tb, Dy, and Y; and heavy rare earth elements (HREE), including Ho, Er, Tm, Yb, and Lu^32–35^.

The test results of thermally affected coals REE values ICP-MS in the Daxing coal mine are shown in Table 6, and the corresponding geochemical parameters are shown in Table 7. (La/Yb)_N_, (La/Sm)_N_, (Gd/Yb)_N_ are the ratios of the standardized values of elemental chondrites. There are three types of enrichment of REE in coal, which are L type (light REE; (La/Lu)_N_ > 1), type M (medium REE; (La/Sm)_N_ < 1, (Gd/Lu)_N_ > 1) and H-type (heavy REE; (La/Lu)_N_ < 1). In coal sample M01, (La/Lu)_N_ is 9.64, (La/Sm)_N_ is 4.24, and (Gd/Lu)_N_ is 1.85, which indicates L-type REE enrichment. Similarly, (La/Yb)_N_ > 1, (La/Sm)_N_ > 1, (Gd/Yb)_N_ > 1 in coal samples M02-M04, it is also enriched for L-type REE.

**Table 6:** Test results of thermally affected coals REE values in the Daxing coal mine.

Elements	Mass fraction of rare earth elements /(µg/g)														
	La	Ce	Pr	Nd	Sm	Eu	Gd	Tb	Dy	Ho	Er	Tm	Yb	Lu	Y
M01	6.57	14.0	1.50	5.75	1.00	0.20	1.05	0.15	0.91	0.17	0.46	0.075	0.45	0.068	5.45
M02	5.27	10.4	1.16	4.53	0.84	0.20	0.80	0.12	0.74	0.14	0.44	0.065	0.40	0.066	4.81
M03	3.53	6.91	0.80	3.24	0.67	0.16	0.67	0.12	0.71	0.14	0.36	0.054	0.33	0.053	4.94
M04	3.25	7.17	0.90	3.76	0.85	0.16	0.97	0.16	1.00	0.21	0.53	0.078	0.48	0.070	7.85
Average values	4.66	9.62	1.09	4.32	0.84	0.18	0.87	0.14	0.84	0.17	0.45	0.07	0.42	0.06	5.76
Chondrites	0.361	0.960	0.134	0.714	0.233	0.088	0.301	0.056	0.380	0.085	0.249	0.036	0.240	0.036	2.200

**Table 7:** The thermally affected coals REE contents and geochemical parameters in the Daxing coal mine.

Coal samples	LREE/ (µg/g)	MREE/ (µg/g)	HREE/ (µg/g)	∑REE/ (µg/g)	LREE/ HREE	(La/Lu)_N_	(La/Sm)_N_	(Gd/Lu)_N_	δEu	δCe
M01	27.82	8.76	1.22	37.80	22.80	9.64	4.24	1.85	0.59	1.02
M02	21.36	7.51	1.11	30.00	19.24	7.96	4.05	1.45	0.74	0.96
M03	14.48	7.27	0.94	22.69	15.40	6.64	3.40	1.51	0.72	0.94
M04	15.08	10.99	1.37	27.41	11.01	4.63	2.47	1.66	0.53	0.96
Average values	19.69	8.63	1.16	29.48	17.11	7.21	3.54	1.62	0.65	0.97

**Figure 9:**
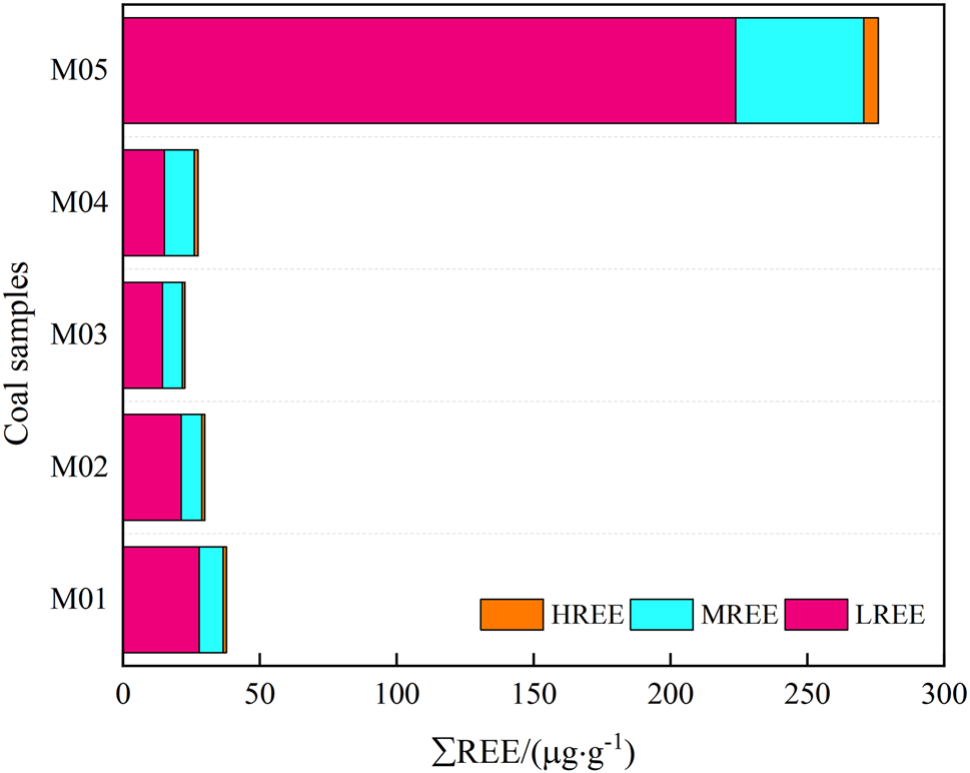
The contents of REE in coal samples.

The total amount of REE in thermally affected coals of the Daxing coal mine is relatively low. It can be seen from Table 7 that ΣREE is 22.69–37.8 μg/g, with an average of 29.48 μg/g. The contents of rare earth elements are variable in the samples of different mines, different coal seams and single coal seam of Daxing coal mine, and the coal seams in this study area are greatly affected by magmatic intrusion. Compared with the unaffected coals in other coalfields, the contents of REE in the study area appears to be depleted. Among them, the contents of LREE ranges from 14.48 to 27.82 μg/g, with an average of 19.69 μg/g. The contents of MREE ranges from 7.27 to 10.99 μg/g, on average 8.63 μg/g. HREE contents are 0.94–1.37 μg/g, with an average of 1.16 μg/g. Among them, LREE/HREE is 11.01–22.8, with an average of 17.11, which is characterized by LREE enrichment and HREE deficit. It can be seen in Fig. 9 that the contents of REE in coal samples affected by magma intrusion are obviously different from that in coal samples not affected by magma intrusion.

**Figure 10:**
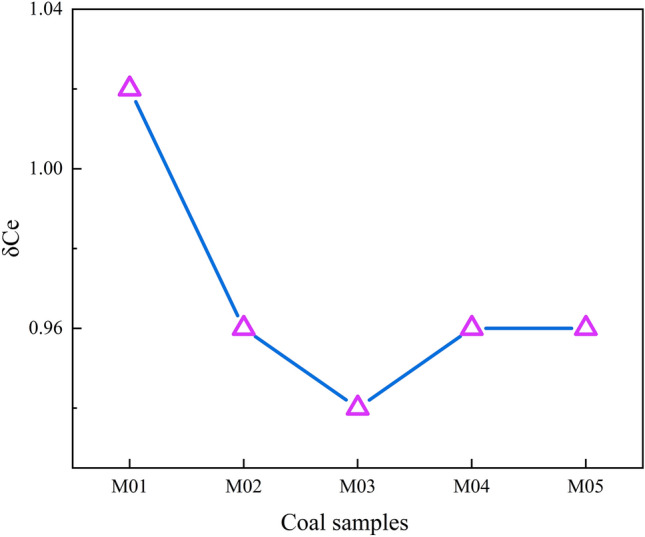
The variation curve of δCe in coal samples from the Daxing coal mine.

In thermally affected coals Eu are distributed between 0.53 and 0.74, with an average of 0.65. The negative Eu is obviously abnormal, as shown in Fig. 10. Ce is distributed in 0.94–1.02, with an average of 0.97, less than 1, which means that Ce is negative anomaly. Generally, the negative anomaly of Ce is mainly caused by the following factors: seawater erosion, sedimentary source area and volcanic hydrothermal solution. Under alkaline conditions, because the water in the sediment is rich in oxygen, Ce^3+^ is oxidized to Ce^4+^, showing a negative abnormality of Ce^36^. Since there was no seawater influence in the palaeomires of the Daxing coal mine, the negative abnormality of Ce may be due to the intrusion of magma and some material exchange in contact with natural coke that leads to the negative abnormality of Ce^37^.

**Figure 11:**
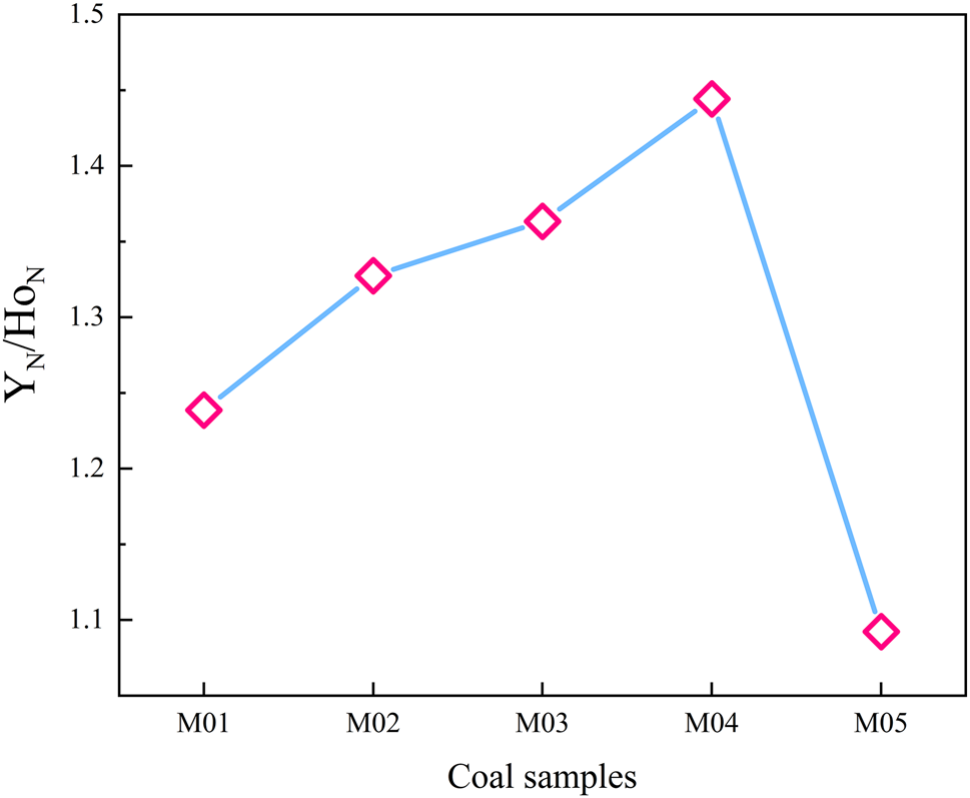
The variation curve of Y_N_/Ho_N_ ratio in coal samples from the Daxing coal mine.

The ratio of Y_N_ to Ho_N_ reflects the Y anomaly in the REE pattern. There are many causes of Y anomalies in coals, mainly geochemical processes in sediment source rocks, sedimentary environments (such as seawater injection) and hydrothermal fluids^38–41^. As shown in Fig. 11, the Y_N_/Ho_N_ in the thermally affected coals in the study area is between 1.24 and 1.44, with an average of 1.34, which shows a positive anomaly of Y. The peneration of hydrothermal solution is one of the factors leading to the positive Y anomaly in the coals. The study of Ge-rich coal in the Ulantuga deposit of Shengli Coal found that after experiencing magma intrusion, the Y content of high Ge-bearing coal is significantly higher than that of low Ge bearing coal in the same coal field^42^. The coal samples M01–M04 in the study area show Y-positive anomalies after being intruded by igneous rocks.

**Figure 12:**
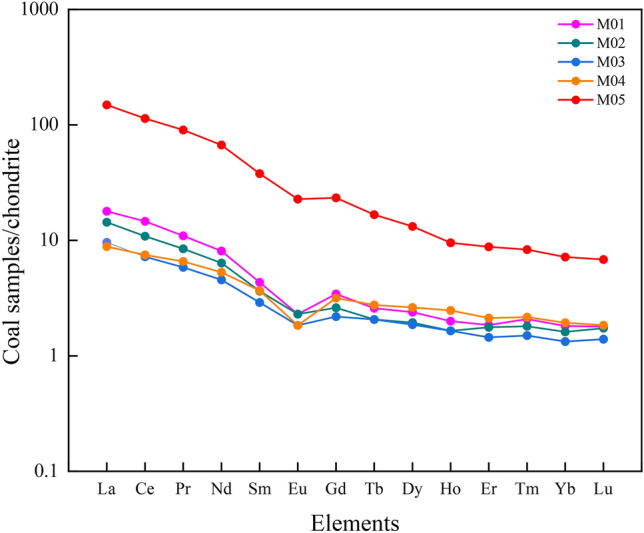
Distribution pattern of REE in coal samples from the Daxing coal mine.

The standardized distribution pattern of rare earth element chondrites in the coal seams of Daxing coal mine are shown in Fig. 12. It can be seen that the distribution pattern of REE in the coal seams of Daxing coal mine is similar, which is a “V ” curve of negative Eu anomaly. The degree of fractionation between LREE and HREE can be reflected by the slope of the distribution model curve between La-Y. It can also be seen intuitively from Fig. 12 that the fractionation degree between HREE is low, while that between LREE is high^43–45^. According to the distribution pattern of REE in the five coal samples collected, the sources of REE in the coal seams of Daxing coal mine are consistent in the peat forming stage, and the supply of terrigenous materials is relatively stable^46^.

## Conclusions

In this paper, X-ray fluorescence spectroscopy and inductively coupled plasma mass spectrometry were combined to study the changes of industrial analysis components and the geochemical characteristics of the major oxides, trace elements and REE of coals in the Daxing coal mine after magma intrusion, and the main conclusions are as follows:

1. Compared with the same coal seams and samples on the same mining face, it was found that the moisture and volatile matter contents of thermally affected coals were lower than that of normal coals, and the moisture and volatile matter content were lower the closer the distance from the magma intrusion.
2. During the magma intrusion process, some minerals remain on the surface of the coal body, resulting in changes in the mineral composition of the coal body. With the distance between the original carbon subnets decreasing, the degree of microcrystalline aggregation and ductility increased, and the aromatity increased, indicating that the intrusion of magma played a positive role in the improvement of the degree of coals metamorphism.
3. The thermally affected coals with different degrees of influenced from magma intrusion have different distribution laws of the major oxides. The closer to the magma intrusion zone, the easier the major oxides are to lose. However, magma intrusion does not make all major oxides disappear, and there are also a small number of enrichments, such as CaO and MnO.
4. The contents of thermally affected coals REE in the Daxing coal mine are low with a similar distribution pattern, showing a wide “V ” curve with high left and low right, which showed the characteristics of LREE enrichment.

## Data Availability

The data and materials presented in this study are available on request from the corresponding author.
